# Genetic support of causal association between lipid and glucose metabolism and stress urinary incontinence in women: a bidirectional Mendelian randomization and multivariable-adjusted study

**DOI:** 10.3389/fendo.2024.1394252

**Published:** 2024-09-16

**Authors:** Nanyan Xiang, Shiqi Su, Yong Yang, Yurui Luo, Tingting Fu, Le Wang, Yifei Lin, Jin Huang

**Affiliations:** ^1^ Department of Urology, Innovation Institute for Integration of Medicine and Engineering, Frontiers Science Center for Disease-Related Molecular Network, West China Hospital, Chengdu, Sichuan, China; ^2^ Health Management Center, General Practice Medical Center, Innovation Institute for Integration of Medicine and Engineering, West China Hospital, Sichuan University, Chengdu, Sichuan, China; ^3^ Key Laboratory of Bio-Resource and Eco-Environment of Ministry of Education, College of Life Sciences, Sichuan University, Chengdu, Sichuan, China; ^4^ Program in Genetic Epidemiology and Statistical Genetics, Department of Epidemiology, Harvard T.H. Chan School of Public Health, Boston, MA, United States

**Keywords:** stress urinary incontinence, lipid, glucose, Mendelian randomization, genetic epidemiology

## Abstract

**Background:**

Stress urinary incontinence (SUI) is a common condition characterized by urethral sphincter failure and urine leakage. Its prevalence in women is higher than in men, and estimates of crude prevalence rates vary widely due to factors such as research methodologies, study populations, and underreporting by patients. This variability hinders research and impacts patient diagnosis, treatment, and quality of life. The complex etiology of SUI is not fully understood, and previous studies have primarily focused on non-invasive indicators. While emerging observational research suggests a correlation between SUI in women and abnormalities in lipid and blood metabolism, the underlying biological mechanisms and causal relationships require further investigation. This study aims to explore the causalities between SUI in women and lipid and blood metabolism.

**Methods:**

Using bidirectional univariate Mendelian randomization (MR), we investigated the causal association between SUI liability in women (case/control = 5,924/399,509) from UK Biobank and lipid and glucose metabolism, indicated by total cholesterol (TC, *N* = 61,166), low-density lipoproteins (LDL, *N* = 58,381), high-density lipoproteins (HDL, *N* = 60,812), triglycerides (TG, *N* = 60,027), fasting glucose (FG, *N* = 19,745), and fasting insulin (FI, *N* = 38,238) from ENGAGE consortium. To account for potential confounding effects, multivariable MR (MVMR) analyses were performed, adjusting for body mass index (BMI) and separately among lipid and glucose metabolism.

**Results:**

We found that increased genetically proxied TC, LDL, and HDL levels were associated with an elevated risk of SUI in women (OR: 1.090–1.117, all *P* < 0.05), These associations were further supported by MVMR analyses with adjustment for BMI (OR: 1.087–1.114, all *P* < 0.05). Conversely, increased FG and FI were associated with reduced SUI reliability in women (OR: 0.731–0.815, all *P* < 0.05). When adjusting among lipid and glucose metabolism, only HDL and FI demonstrated causal effects. Reverse MR analyses provided no genetic evidence supporting the causal effect of SUI in women on lipid and blood metabolism (all *P* > 0.05).

**Conclusions:**

Our results reported that increased TC, LDL, and HDL are linked to higher SUI susceptibility in women, while higher FG and FI levels have a protective effect. In overweight/obese women with metabolic abnormalities, the positive associations between TC, LDL, and HDL levels and SUI indicate a higher risk.

## 1 Introduction

Urinary incontinence (UI) is characterized by the frequent urge to urinate or involuntary leakage of urine. Stress urinary incontinence (SUI) is a common form of urinary incontinence characterized by the failure of the urethral sphincter to close completely, resulting in urine leakage (1). Due to factors such as pregnancy, childbirth, and hormonal changes during menopause, the incidence of SUI in women is higher than in men (2). It was reported that SUI occurs at least weekly in one-third of adult women (3). Estimates of its crude prevalence rate in the female population range from 10% to 40%, which have reported significant variations (4, 5). On the one hand, it attributed to differences in research methodologies, study populations, and definitions of UI (6). On the other hand, given the sensitive and potential nature of UI, patients may tend to underreport their symptoms, resulting in an underestimated prevalence and a reluctance to seek medical assistance (7). This wide variability not only hinders relevant research but also has significant implications for the diagnosis, treatment, management, health status, and overall quality of life of patients.

The etiology and pathogenesis of SUI are complex and not yet completely clear. Prior research on the etiology or risk factors of SUI have primarily focused on indicators that are available by easy screening methods, such as BMI, weight, pregnancy/childbirth history, and history of uterine/bladder disease treatment (8–10). Notably, a growing number of researchers are finding that SUI frequently accompanies abnormalities in blood lipid and glucose-related biomarkers. Among women with obesity, hyperlipidemia was associated with a higher risk of SUI, and there was a significant synergistic effect of hyperlipidemia and obesity on SUI (11). Furthermore, a cross-sectional study showed that the levels of blood glucose were positively correlated with SUI (12). However, the precise biological mechanism underlying this reciprocal association and the establishment of causal inference remain inadequately understood due to the effect of confounding factors and the absence of randomized controlled trial (RCT) studies.

Mendelian randomization (MR) is an epidemiological technique to estimate the causal associations, utilizing genetic variants as instrumental variables which are not expected to be subject to biases from confounding and reverse causation seen in other types of observational epidemiology (13, 14). To investigate a clearer causal effect of lipid and glucose metabolism on SUI in women, we performed bidirectional two-sample MR analysis and multivariable MR (MVMR) analysis using 405,433 European ancestry participants. Furthermore, we also adjusted the confounding effect of BMI in the causal inference of lipid and glucose metabolism on SUI in women using MVMR.

## 2 Materials and methods

### 2.1 Study design and populations

**Figure 1:**
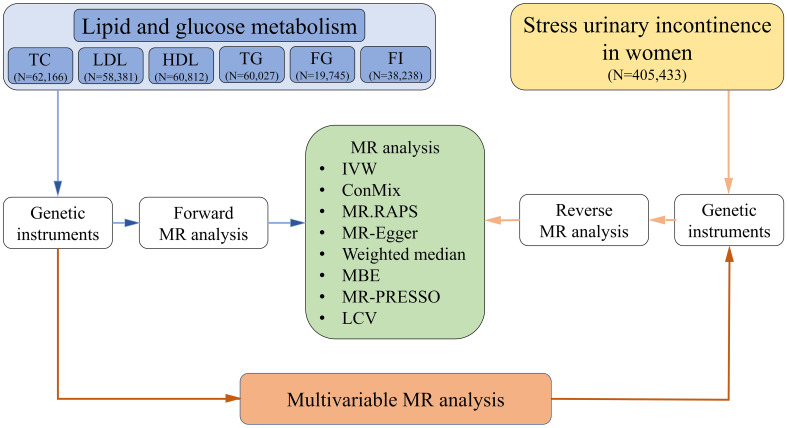
Flow chart. TC, total cholesterol; LDL, low-density lipoproteins; HDL, high-density lipoproteins; TG, triglycerides; FG, fasting glucose; FI, fasting insulin; MR, Mendelian randomization; IVW, inverse-variance weighted method; ConMix, contamination mixture; RAPS, robust adjusted profile score; MBE, mode-based estimate; PRESSO, pleiotropy residual sum and outlier; LCV, latent causal variable.

We utilized bidirectional two-sample Mendelian randomization (MR) (15) to assess the causal association between lipid and glucose metabolism and SUI in female patients. We ensured that the study sample is derived from a relatively genetically homogenous population (European ancestry). **Figure 1** shows an overview of the study design.

Regarding lipid and glucose metabolism biomarkers, all the summary statistical data were continuous biomarkers from the European Network for Genetic and Genomic Epidemiology (ENGAGE) consortium (16, 17), including total cholesterol (TC, *N* = 61,166), low-density lipoproteins (LDL, *N* = 58,381), high-density lipoproteins (HDL, *N* = 60,812), triglycerides (TG, *N* = 60,027), fasting glucose (FG, *N* = 19,745), and fasting insulin (FI, *N* = 38,238). According to summary statistics, SUI in women was found in a total of 405,433 individuals (case/control = 5,924/399,509), derived from UK Biobank/PheWeb (18). Moreover, genetic association estimates for BMI were also obtained from 346,393 European ancestry individuals in UK Biobank (19). All data of this study are publicly available GWAS summary statistics. Therefore, additional ethical approvals for the GWAS were not required.

### 2.2 Selection of genetic instrumental variable

For forward MR, instrumental variables were identified as genetic variants associated with the abovementioned six lipid and glucose metabolism biomarkers. To meet the first assumption of MR that the instrumental variables are strongly associated with exposures (lipid and glucose metabolism), conditionally independent SNPs at a level of genome-wide significance threshold (*p* < 5 × 10^−8^) were selected. For FI, only one SNP was selected with the threshold of *p* < 5 × 10^−8^, so we set the *p*-value to 5 × 10^−5^ to get adequate SNPs for analysis. All genetic variants were clumped using PLINK to ensure that a list of lipid and glucose metabolism SNPs was from an independent set of variants (parameters: –clump-p1 5 × 10^−8^ –clump-p2 1 × 10^−5^ –clump-r2 0.1 –clump-kb 1000). For reverse MR (testing the effect of genetic predictors of SUI in women on lipid and glucose metabolism), we applied the *p*-value threshold of *p* < 5 × 10^−6^ to SUI in women. The independent instruments were determined by clumping (parameters: –clump-p1 5 × 10^−6^ –clump-p2 1 × 10^−5^ –clump-r2 0.1 –clump-kb 1000).

For the third assumption, there should be no SNP associated with unobserved confounding variable and outcomes (20). We performed a sensitivity analysis with a *p*-value threshold of *p* < 5 × 10^−10^ in lipid and glucose metabolism SNPs (set *p* < 5 × 10^−6^ for FI). In addition, we set *r* ^2^ to 0.001, a more stringent threshold, to reduce the risk of horizontal pleiotropy. For the reverse analysis, to include adequate SNPs for the analysis, we applied a more relaxed *p*-value threshold of *p* < 5 × 10^−5^.

### 2.3 Statistical analyses

In order to provide unbiased estimates for Mendelian randomization, three key assumptions should be met: (1) genetic instrumental variants should be significantly associated with the exposure (i.e., lipid and glucose metabolism), (2) the genetic instrumental variants affect the outcome (i.e., SUI in women) only through the effect of exposure (i.e., lipid and glucose metabolism) indirectly and not through other biological pathways (14), and (3) genetic instrumental variants should be independent of any confounders. This study adhered to the Strengthening the Reporting of Observational Studies in Epidemiology—Mendelian Randomization guidelines (STROBE—MR) (21).

To investigate the potential causal relationship between lipid and glucose metabolism and SUI in women, we applied several MR methods. First, inverse-variance weighted (IVW) was performed. It is a common MR approach, which assumed that all instruments were valid and could be liable to be biased with any instrument exhibiting horizontal pleiotropy (22). Thus, for the second assumption, the primary analysis method in this study was the mode-based estimate (MBE) method, which allows the majority of instruments to be pleiotropic (23). To minimize the effect of horizontal pleiotropy, weighted median analysis (24) and MR-Egger regression (25) were also performed. Contamination mixture (ConMix) (24) and robust adjusted profile score (RAPS) (26) were performed to detect the causal effect of invalid instruments’ bias, pleiotropy, and extreme outliers. In addition, we used the pleiotropy residual sum and outlier (PRESSO) method to detect horizontal pleiotropy and correct for horizontal pleiotropy via outlier removal (27).

We introduce a latent causal variable (LCV) model, which is robust to sample overlap. Under this model, the genetic correlation between two traits is mediated by a latent variable having a causal effect on each trait (28). Moreover, MVMR is an MR extension analogue to estimate the effect of two or more correlated exposure variables (29). Thus, we applied MVMR based on the IVW methods separately for lipid and glucose metabolism biomarkers, which are closely related risk factors on the same molecular level. As BMI was reported to be a factor that affects the metabolic levels of lipid and glucose, we additionally applied MVMR based on the IVW methods to adjust BMI-associated variants.

All analyses were performed with R software 4.2.2. ConMix, IVW, weighted median, MBE, MR–Egger methods, and multivariable MR were performed using the “MendelianRandomization” package. The RAPS model was performed using the “mr. raps” package. The MR-PRESSO approach was performed using the “MR-PRESSO” package. We used the mRnd tool (https://shiny.cnsgenomics.com/mRnd/) to calculate the F-statistics to assess the strength of the instruments (30). F-statistic greater than 10 was considered a strong instrument (31).

### 2.4 Ethics approval

This MR study was based on publicly available GWAS data, obviating the need for ethical approvals.

## 3 Results

### 3.1 Univariable MR

#### 3.1.1 Effects of lipid and glucose metabolism on SUI in women

In the MR analysis of lipid and glucose metabolism on SUI in women, the total number of genetic instruments retained in terms of genome-wide threshold (*p* < 5 × 10^−8^, *r* ^2^ < 0.1) from the ENGAGE consortium was 151, 149, 133, 93, and 28 SNPs for TC, LDL, HDL, TG, and FG, respectively. Setting the *p*-value to 5 × 10^−5^, we obtained 152 instrumental SNPs for FI. F-statistics across SNPs ranged from 997 to 7,223 among lipid and glucose metabolism biomarkers, which are displayed in **Supplementary Table S1**.

**Figure 2:**
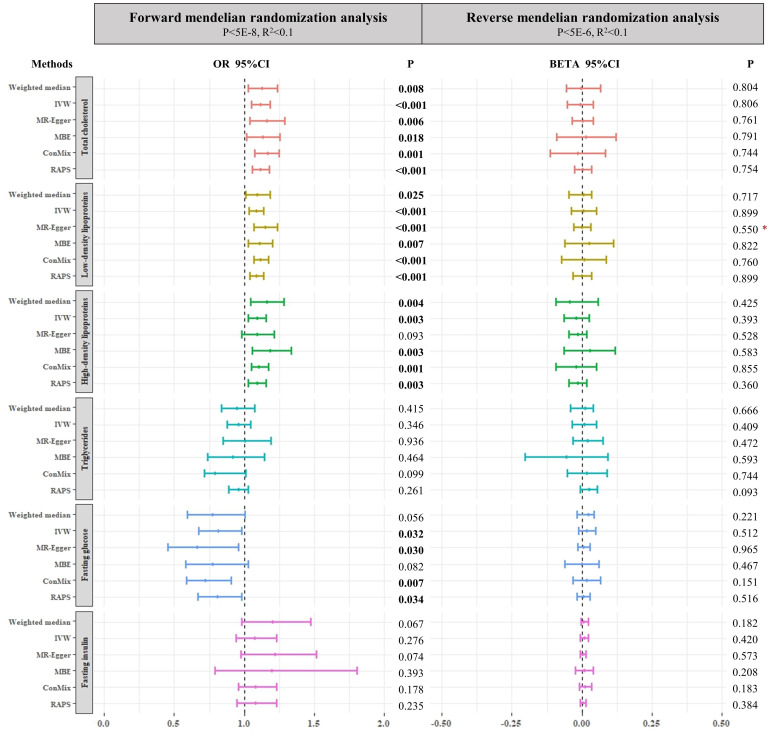
Forest plots of main forward and reverse Mendelian randomization analyses investigating the causal relationships between both lipid and glucose metabolism biomarkers and SUI in women. The p-value threshold of forward Mendelian randomization for fasting insulin is 5E-5. Asterisk denotes that the intercepts of MR–Egger were significant (*p* < 0.05). IVW, inverse-variance weighted method; ConMix, contamination mixture; RAPS, robust adjusted profile score; MBE, mode-based estimate.

For lipid metabolism biomarkers, IVW, MBE, ConMix, RAPS, and weighted median analyses all identified significant positive associations between TC/LDL/HDL and SUI in women. MR-Egger regression also suggested the positive effect of TC and LDL with no significant intercept (all OR >1, *P* < 0.05). There is no evidence of causal relationship between TG and SUI in women (all *P* > 0.05). For glucose metabolism biomarkers, a significant negative causal effect of FG on the risk of SUI was identified in female patients (IVW OR = 0.815, 95%CI: 0.676–0.981, *P* = 0.032; the results of MR-Egger, ConMix, and RAPS are aligned), while we found no significant causal relationship between FI and SUI in women when using the threshold of *p* < 5 × 10^−5^, *r* ^2^ < 0.1 to select SNPs of FI (all *P* > 0.05) (**Figure 2** and **Supplementary Table S2**).

As we adjusted the genome-wide threshold of *p* < 5 × 10^−10^ and *r* ^2^ < 0.1, a total of 99, 112, 87, 56, and 15 instrumental SNPs was selected for TC, LDL, HDL, TG, and FG, respectively. For lipid metabolism biomarkers, we also found some similar causal effect of TC, LDL, and HDL on SUI in women (all OR >1, *P* < 0.05). Furthermore, the ConMix method found an additional causal role of genetically decreased risk of SUI in female patients with elevated TG (OR = 0.798, *P* = 0.01). For glucose metabolism biomarkers, in addition to the negative causal association between FG and SUI in women (IVW OR = 0.7, 95%CI: 0.538–0.9112, *P* = 0.008; RAPS: OR = 0.699, 95%CI: 0.5358–0.9129, *P* = 0.009), we also found a negative effect of FI on SUI in women with 24 instrumental SNPs selected by the threshold of *p* < 5 × 10^−6^ and *r* ^2^ < 0.1 (ConMix OR = 0.460, 95%CI: 0.248–0.909, *P* = 0.030; the results of IVW and RAPS are aligned) (**Supplementary Table S2**).

After setting the *R* ^2^ as 0.001, we obtained 59 SNPs for TC, 53 SNPs for LDL, 56 SNPs for HDL, 36 SNPs for TG, 17 SNPs for FG (genome-wide threshold *p* < 5 × 10^−8^, *r* ^2^ < 0.001), and 143 SNPs for FI (genome-wide threshold *p* < 5 × 10^−5^, *r* ^2^ < 0.001). Genetically predicted higher TC/HDL was associated with an increased risk of SUI in women. With the more stringent threshold (TC, LDL, HDL, TG, and FG: *p* < 5 × 10^−10^/FI: *p* < 5 × 10^−6^, *r* ^2^ < 0.001), we found that a positive causal effect of genetically predicted TC (45 SNPs)/LDL (41 SNPs)/HDL (33 SNPs) (all OR >1, *P* < 0.05) and a negative causal effect of FG (25 SNPs)/FI (24 SNPs) (all OR <1, *P* < 0.05) were also significant. For significant TG-related instrumental SNPs, we did not find evidence of a causal effect on SUI in women under any genome-wide thresholds (*p* < 5 × 10^−8^/*p* < 5 × 10^−10^, *r* ^2^ < 0.001) (**Supplementary Table S3**).

The MR-PRESSO process had verified all the causal associations mentioned above (**Supplementary Table S4**).

#### 3.1.2 Effects of SUI in women on lipid and glucose metabolism

We obtain 21 SNPs for SUI in women without effects of linkage disequilibrium (*r* ^2^ < 0.1), which reached the primary genome-wide significant level (*p* < 5 × 10^−6^). To retain more instrumental SNPs and avoid over-inflated type I errors in these reverse MR analyses, we also performed analysis by setting genome-wide significance of *p* < 5 × 10^−5^. Non-significant causal association was found in all analyses (all *P* > 0.05) (**Figure 2** and **Supplementary Table S5**). Furthermore, MR-PRESSO analyses also did not find any significant associations (**Supplementary Table S6**).

### 3.2 LCV

The LCV method was employed to investigate if the causal effect of lipid and glucose metabolism on SUI in women could be fully explained by shared underlying genetic etiology. Our finding identified evidence for genetic positive correlations between TC, LDL, and HDL and SUI (Rho = 0.11–0.22, all *P* < 0.05) with partial causal effect (GCP = 0.68–0.77). For glucose metabolism biomarkers, we only obtained one negative genetic effect of FI through LCV analysis [Rho = -0.12 (0.17), GCP = 0.77 (0.08), *P <*0.001] (**Table 1**).

**Table 1:** Results of LCV modeling for the genetic relationships between both lipid and glucose metabolism biomarkers and SUI in women.

Trait 1	Trait 2	P _LCV_	GCP (std err)	Rho (std err)
SUI in women	TC	8.03E-08	0.68(0.22)	0.13(0.07)
	LDL	5.18E-59	0.77(0.09)	0.11(0.09)
	HDL	1.48E-10	0.77(0.16)	0.22(0.08)
	TG	0.624	0.12(0.56)	-0.06(0.07)
	FG	0.306	0.36(0.24)	0.04(0.10)
	FI	5.65E-11	0.77(0.08)	-0.12(0.17)

Rho represents the genetic correlation between the two traits, estimated by LD score regression. GCP is an estimate of the genetic component of trait 1 which is causal for trait 2. A value closer to 1 represents stronger causality of trait 1 on trait 2.

TC, total cholesterol; LDL, low-density lipoproteins; HDL, high-density lipoproteins; TG, triglycerides; FG, fasting glucose; FI, fasting insulin; SUI, stress urinary incontinence.

### 3.3 Multivariable MR

We performed MVMR separately for lipid and glucose metabolism biomarkers to disentangle the causal effect on SUI in women. Only HDL retained a positive causal relationship with SUI in women while controlling TC, LDL, and TG in the model (BETA = 0.232, 95%CI: 0.054–0.411, *P* = 0.011). When the independent causal effects of FG and FI were evaluated together in one MVMR, only FI showed a negatively causal effect on SUI in women (BETA = -0.383, 95%CI: -0.678–0.089, *P* = 0.011) (**Table 2**).

**Table 2:** Causal relationships of lipid and glucose metabolism biomarkers on SUI in women estimated by multivariable MR, adjusting for BMI and mutual adjustments among lipid biomarkers and among glucose biomarkers.

Instrumental SNPs	Exposures	Outcome	MVMR-IVW Beta (95%CI)	*P*
129	TC	SUI in women	0.097(0.021, 0.174)	0.013
	BMI		0.145(-0.273, 0.563)	0.497
123	LDL	SUI in women	0.083(0.018, 0.148)	0.012
	BMI		0.131(-0.267, 0.529)	0.52
118	HDL	SUI in women	0.108(0.037, 0.180)	0.003
	BMI		0.131(-0.248, 0.529)	0.502
91	TG	SUI in women	-0.038(-0.148, 0.072)	0.503
	BMI		0.165(-0.281, 0.661)	0.469
48	FG	SUI in women	-0.155(-0.418, 0.107)	0.245
	BMI		-0.039(-0.552, 0.415)	0.781
104	FI	SUI in women	-0.088(-0.323, 0.147)	0.464
	BMI		0.033(-0.392, 0.459)	0.878
419	TC	SUI in women	-0.33(-0.784, 0.123)	0.154
	LDL		0.376(-0.019, 0.771)	0.062
	HDL		0.232(0.054, 0.411)	0.011
	TG		0.064(-0.101, 0.228)	0.448
51	FG	SUI in women	-0.117(-0.303, 0.068)	0.215
	FI		-0.383(-0.678, -0.089)	0.011

TC, total cholesterol; LDL, low-density lipoproteins; HDL, high-density lipoproteins; TG, triglycerides; FG, fasting glucose; FI, fasting insulin; SUI, stress urinary incontinence.

As BMI could affect lipid and glucose metabolism, as well as SUI in women, we established MVMR models to account for the influence of BMI-associated variants. Regarding lipid metabolism biomarkers, the positive associations for genetically predicted levels of TC/LDL/HDL remained significant in multivariable MR analyses with adjustment for genetically predicted levels of BMI (TC BETA = 0.097, 95%CI: 0.021–0.174, *P* = 0.013; LDL BETA = 0.083, 95%CI: 0.018–0.148, *P* = 0.012; HDL BETA = 0.108, 95%CI: 0.037–0.180, *P* = 0.003). However, in contrast to univariable MR analyses, multivariable MR presented no corresponding associations between both FG and FI and SUI in women after adjustment for BMI (FG BETA = -0.155, 95%CI: -0.418–0.107, *P* = -0.245; FI BETA = -0.088, 95%CI: -0.323–0.147, *P* = 0.464) (**Table 2**).

## 4 Discussion

### 4.1 Main findings

In this study, we applied bidirectional two-sample MR method to explore the causal association of lipid (TC, LDL, HDL, and TG) and glucose (FG and T2D) metabolism traits with SUI in women based on summary-level data from large-scale datasets. The primary analyses revealed that genetically predicted SUI in women was associated with a wide range of lipid and glucose metabolism biomarkers. Specifically, we observed significant positive associations between the levels of TC, LDL, and HDL and the risk of SUI in women. Furthermore, we observed a protective causal effect of FG and FI levels on SUI liability in women. Reverse MR analyses provided genetic evidence supporting a deleterious causal effect of SUI in women on increased levels of TG. Additionally, after adjusting for BMI, the causal effect of TC, LDL, and HDL on SUI in women was supported in the multivariable MR analysis, while separate MVMR analyses among lipid and glucose metabolism biomarkers only demonstrated significant effects of HDL and FI.

Standard lipid blood tests consist of four basic parameters: TC, LDL, HDL, and TG. In our study, we found that the higher levels of TC, LDL, and HDL had a genetic causal effect on SUI liability in women, while there was no significant relationship between TG and SUI in women, which was also supported by LCV analysis and MVMR analysis adjusting BMI. However, this finding differed from the conclusions of previous observational studies. A study had shown that cholesterol and triglycerides have no significant effect on the risk of urinary incontinence in women with diabetes mellitus (32), while another had reported that TG was higher in older women with SUI than in age- and sex-matched control groups without clinical SUI (33). We believe that these differences may originate from the lack of adjustment for certain covariates. Therefore, we conducted MVMR analysis, mutually adjusting among lipid biomarkers that are closely related risk factors at the same molecular level. After adjustment, only HDL retained a positive causal correlation with SUI.

Generally, it is believed that high levels of LDL, which are associated with an increased risk of multisystem disorders, can cause cholesterol to stick to the walls of blood vessels and contribute to plaque formation (34). In contrast, HDL carries cholesterol from the blood vessels to the liver for absorption and can counter the effects of LDL (35). Interestingly, in our analyses, elevated LDL and HDL levels were both associated with SUI liability in women. On the one hand, as we have discussed above, we believed that some potentially complex confounding factors in the causal relationship between LDL and SUI are important reasons for this unusual result (36). On the other hand, extreme high HDL concentrations may paradoxically have deleterious effects on health status (37)—for instance, certain genetic variants associated with extreme high HDL cholesterol concentrations have been paradoxically related with the risk of cardiovascular disease (38, 39). Further targeted investigations and clinical trials are warranted to explore the different effects on SUI in women across a range of HDL concentrations as well as to identify the specific pathways and molecular targets that mediate the relationship between different HDL levels and SUI, which could inform additional therapeutic development.

Some literatures have discussed the physiological mechanisms by which lipid metabolism affects pelvic floor structure and function. A study on animals demonstrated that prolonged exposure to the dyslipidemic environment triggers epigenetic changes, resulting in aberrant global transcriptional signatures of genes and microRNAs (40, 41). This process impairs the reparative capacity of muscle-derived stem cells, consequently impacting the recovery of pelvic floor function, leading to SUI in women (41, 42). Mohamed Kamal Mesregah et al. found that the rate of incontinence was higher in patients with chronic hyperlipidemia with significantly increased nerve root injury (43). Therefore, we thought that lipid metabolism biomarkers could play an important role in the identification of pelvic floor nerve injury. It suggested that clinicians could include these lipid biomarkers in examination when assessing the risk of SUI in women. Additionally, for women with a family history of SUI (44), clinicians should monitor their lipid levels more frequently and intervene in cases of abnormalities to prevent the occurrence of SUI earlier.

Regarding glucose metabolism, there were some evidence supporting a causal effect of a lower level of FG on high risk of SUI in women, which was opposite to the conclusion that Jeanette S. Brown et al. reported. In their investigation of 1,461 nonpregnant adult women, they found that the prevalence of incontinence was significantly higher in women with high glucose levels than those with normal FG (45). Moreover, we observed a significantly low level of FI in SUI female patients with a more relaxed *p*-value threshold (*p* < 5 × 10^−6^), which was supported by our LCV analysis and MVMR analysis adjusting for FG. As BMI or obesity, often accompanied by hyperglycemia and insulin resistance (46, 47), is widely recognized as a significant risk factor for SUI in women, we also conducted MVMR analysis adjusting BMI for glucose biomarkers. However, the results revealed no significant association between SUI in women and FG and FI levels. This underscores the limitations of using glucose-related biomarkers as independent predictors of SUI (51) despite high glucose being considered a risk factor for UI. Considering BMI as a potential modifiable risk factor, amenable to lifestyle modifications, underscores the importance of integrating clinical and lifestyle interventions in the future management of SUI in women (48). By employing comprehensive interventions, such as combining dietary control, increased physical activity, and medication therapy, along with improving patients’ weight and blood glucose levels, the risk of SUI may be more effectively reduced.

### 4.2 Strength and limitation

This present study was the first to demonstrate the causal associations between lipid and glucose metabolism and SUI in women using MR analysis based on largest GWAS data. The various MR methods that we utilized guaranteed the reliability of results by eliminating residual confounding as much as possible. Meanwhile, given the effect of BMI and mutual interaction of metabolism traits, we applied MVMR to adjust these exposures. In addition, the F-statistics of the genetic variants were all more than 10 with a large sample size, which indicated that the genetic variants were strong enough to be IVs for exposure.

However, there were some limitations that need to be acknowledged. Firstly, we did not differentiate between the levels of lipid and glucose metabolism biomarkers in our analyses. Despite the fact that the potential impact on our results is unclear, we assumed the paradoxically causal direction of extreme high HDL and proposed a new and significant research aspect for SUI in women. Secondly, our study population was limited to individuals of European ancestry, and thus caution should be taken when generalizing the results to populations of other ancestries (49).

## 5 Conclusion

In conclusion, our MR estimates supported an adverse effect of increased TC/LDL/HDL and a protective effect of increased FG and FI on SUI susceptibility in women. The positive associations between TC/LDL/HDL and SUI in women were also verified through MVMR models with adjusted BMI, which revealed the risk of SUI in obese/overweight women with metabolic abnormalities. The potential mechanism of the causal relationship between lipid and glucose metabolism and SUI warrants further investigations.

## Data Availability

The original contributions presented in the study are included in the article/**Supplementary Material**. Further inquiries can be directed to the corresponding authors.
